# Clinicopathological Characteristics, Tumor Marker Profile, and Hepatic Metastasis Patterns in Colorectal Cancer: A Retrospective Observational Study at a Regional Oncology Center in the Dominican Republic (2018-2022)

**DOI:** 10.7759/cureus.106184

**Published:** 2026-03-31

**Authors:** Franklin M Valerio Gonzalez, Adrian Ureña Gomez, Enmanuel Taveras Alba, Robert Tejada Tio, Naly A Cruz

**Affiliations:** 1 Department of Medicine, School of Medicine, Universidad Tecnológica de Santiago, Santiago, DOM; 2 Department of Oncology, Regional Oncology Institute of Cibao, Santiago, DOM

**Keywords:** ca 19-9, cancer epidemiology, carcinoembryonic antigen, caribbean population, clinical staging, colorectal cancer, hepatic metastasis, rectal cancer, retrospective observational study, tumor markers

## Abstract

Background

Colorectal cancer (CRC) remains a major cause of cancer-related morbidity and mortality worldwide. In developing regions, late-stage presentation continues to contribute to a higher burden of metastatic disease. This study aimed to describe the clinicopathological characteristics, tumor marker profile, and frequency of hepatic metastasis among patients diagnosed with CRC at a regional oncology center in the Dominican Republic.

Methods

A retrospective observational study was conducted, including 96 patients with histopathologically confirmed colorectal cancer diagnosed between January 2018 and May 2022. Demographic characteristics, primary tumor location, clinical stage, tumor marker levels (carcinoembryonic antigen (CEA) and carbohydrate antigen 19-9 (CA 19-9)), and the presence of hepatic metastasis were analyzed. Categorical variables were summarized as frequencies and percentages.

Results

Older age and male sex predominated within the study population. Rectal tumors were the most frequent primary location. A substantial proportion of patients were diagnosed at advanced stages, particularly stages III and IV. Hepatic metastasis occurred exclusively among patients with stage IV disease.

Conclusions

In this study, CRC frequently presented at intermediate or advanced stages and predominantly affected older male patients. These findings highlight the importance of early detection and comprehensive staging strategies to reduce metastatic burden in resource-limited settings.

## Introduction

Colorectal cancer (CRC) is the third most commonly diagnosed malignancy worldwide and remains one of the leading causes of cancer-related mortality [1,2]. According to global cancer statistics, CRC accounts for substantial morbidity and mortality, particularly in aging populations and regions undergoing epidemiological transition [3]. Although screening programs have improved early detection rates in high-income countries, many developing regions continue to report diagnoses at intermediate or advanced stages.

In Latin America and the Caribbean, colorectal cancer incidence has shown an increasing trend over recent decades [4]. This pattern is likely multifactorial and may be related to population aging, epidemiological transition, greater exposure to Westernized dietary and lifestyle risk factors, rising obesity rates, and persistent limitations in access to screening and early diagnosis [4,5]. In our study population, 55.2% of patients were diagnosed at stage III or IV disease, further illustrating the burden of advanced presentation in this setting.

The liver represents the most common site of distant metastasis in CRC due to portal venous drainage from the colorectal region [6]. Hepatic involvement significantly affects prognosis and therapeutic decision-making [7]. Early identification of metastatic disease is therefore essential for optimizing treatment strategies and improving survival outcomes. 

Although carcinoembryonic antigen (CEA) and carbohydrate antigen 19-9 (CA 19-9) are not tumor-specific biomarkers, they are commonly used in clinical practice as adjunctive tools for prognostic assessment, disease monitoring, and follow-up in patients with colorectal cancer [8,9]. However, the distribution of these biomarkers and their relationship with disease stage in Caribbean populations has not been extensively described.

Given the limited published data from the Dominican Republic, this study aims to describe the clinicopathological characteristics, tumor marker profile, and frequency of hepatic metastasis among patients diagnosed with colorectal cancer at a regional oncology center between 2018 and 2022.

## Materials and methods

Study design and setting

A retrospective observational study was conducted at the Instituto Oncológico Regional del Cibao, a tertiary oncology center located in Santiago, Dominican Republic. The study period extended from January 2018 to May 2022.

Study population

The study included 96 patients aged 30 years or older with histopathologically confirmed colorectal adenocarcinoma diagnosed during the study period. Patients were required to have complete medical records documenting tumor location and clinical stage at diagnosis. Cases with incomplete records, missing key clinical or staging information, or duplicate entries were excluded from the analysis.

Data collection

Data were obtained through a systematic review of institutional medical records using a standardized data collection form. Extracted variables included demographic characteristics (age and sex), primary tumor location, clinical stage according to the tumor, node, metastasis (TNM) classification system, tumor marker levels (carcinoembryonic antigen (CEA) and carbohydrate antigen 19-9 (CA 19-9)), presence of hepatic metastasis at diagnosis, and documented complications.

Statistical analysis

Categorical variables were summarized as frequencies and percentages. Statistical analyses were performed using Microsoft Excel (Microsoft Corporation, Redmond, WA, USA).

Ethical considerations

This retrospective study utilized anonymized medical record data without direct patient interaction. Patient confidentiality was maintained in accordance with institutional standards.

## Results

A total of 96 patients with histopathologically confirmed colorectal adenocarcinoma were included in the analysis. The study population was predominantly composed of older adults, with the majority of patients over 60 years of age. A male predominance was observed within the study population (Table 1).

**Table 1:** Demographic characteristics of patients with colorectal cancer (n = 96). Data are presented as frequencies and percentages.

Variable	n (%)
Age 30–40 years	7 (7.29%)
Age 41–50 years	15 (15.63%)
Age 51–60 years	13 (13.54%)
Age >60 years	61 (63.54%)
Male	59 (61.46%)
Female	37 (38.54%)

Regarding tumor distribution, rectal cancer represented the most frequent primary location, followed by tumors of the ascending colon. Distal tumor involvement accounted for a substantial proportion of cases, whereas transverse colon and cecal tumors were less commonly observed (Table 2). These findings suggest a predominance of distal colorectal disease within this regional population.

**Table 2:** Primary tumor location among patients with colorectal cancer (n = 96). Distribution of primary tumor location at diagnosis.

Tumor location	n (%)
Rectum	42 (43.75%)
Ascending colon	18 (18.75%)
Descending colon	9 (9.38%)
Sigmoid colon	11 (11.46%)
Cecum	4 (4.17%)
Transverse colon	1 (1.04%)
Colon and rectum	11 (11.46%)

At diagnosis, advanced stages were common. Stage III was the most frequently identified stage, followed by stage II, while nearly one-quarter of patients presented with stage IV disease. Early-stage diagnoses were comparatively less frequent, indicating a tendency toward intermediate or advanced-stage presentation at initial evaluation (Table 3).

Hepatic metastasis occurred exclusively among patients with stage IV disease. Approximately half of the patients classified as stage IV demonstrated liver involvement at diagnosis, whereas no cases were observed among earlier stages.

Tumor marker analysis demonstrated heterogeneous distributions. CEA levels most commonly fell within the low-to-moderate elevation range; however, markedly elevated values were observed in a considerable proportion of patients. In contrast, most individuals demonstrated CA 19-9 levels within the normal reference range, with elevated levels present in a smaller subset. These findings reflect variability in biomarker expression across the study population (Table 3).

**Table 3:** Clinical stage and tumor marker distribution at diagnosis (n = 96). Distribution of clinical stage and tumor marker levels at the time of diagnosis. CEA: carcinoembryonic antigen, CA 19-9: carbohydrate antigen 19-9.

Variable	n (%)
Stage I	14 (14.58%)
Stage II	29 (30.21%)
Stage III	31 (32.29%)
Stage IV	22 (22.92%)
CEA <2.5 ng/mL	20 (20.83%)
CEA 2.5–5 ng/mL	30 (31.25%)
CEA 6–15 ng/mL	10 (10.42%)
CEA 16–30 ng/mL	6 (6.25%)
CEA >30 ng/mL	21 (21.88%)
CEA not available	9 (9.38%)
CA 19-9 <40 U/mL	62 (64.58%)
CA 19-9 40–80 U/mL	7 (7.29%)
CA 19-9 121–150 U/mL	1 (1.04%)
CA 19-9 ≥300 U/mL	6 (6.25%)
CA 19-9 not available	20 (20.83%)

## Discussion

Colorectal cancer (CRC) remains one of the leading causes of cancer-related morbidity and mortality worldwide, ranking among the top three most commonly diagnosed malignancies and a major contributor to cancer-related deaths globally [10]. The global burden of CRC continues to rise, particularly in low- and middle-income countries where screening implementation remains limited and diagnoses frequently occur at advanced stages. Our retrospective study provides a descriptive overview of patients diagnosed with CRC at a regional oncologic center, highlighting demographic characteristics, tumor distribution, and hepatic metastasis patterns in this study population.

In our study population, the majority of patients were older than 60 years (61 (63.54%)), and males predominated (59 (61.46%)). These findings are consistent with global and regional epidemiological analyses demonstrating a higher incidence of colorectal cancer among older individuals and a modest male predominance, trends that have been consistently observed across diverse population-based studies [3,10,11].

Regarding tumor location, the rectum was the most frequently affected site (42 (43.75%)), followed by the ascending colon (18 (18.75%)). Previous population-based and institutional studies from low- and middle-income settings have reported a substantial proportion of distal and rectal tumors, potentially reflecting differences in screening access and diagnostic patterns [4,5,11]. The predominance of rectal tumors in our population underscores the importance of early detection strategies and appropriate staging.

In this study population, hepatic metastasis was observed exclusively among patients with stage IV disease, reflecting the expected pattern of metastatic presentation in colorectal cancer. This finding aligns with established oncologic principles, as the liver remains the most common site of distant metastasis due to portal venous drainage from the colorectal region [6,7]. In this setting, the observed pattern reinforces the importance of systematic staging and careful metastatic assessment at diagnosis.

The overall hepatic metastasis rate in our study (11.46%) is comparable to previously reported rates in retrospective institutional analyses [12]. Variations across studies may reflect differences in screening implementation, staging at diagnosis, and access to specialized oncologic care. Our findings emphasize the clinical relevance of systematic staging and liver imaging in patients diagnosed with CRC.

Tumor marker analysis demonstrated that CEA levels between 2.5 and 5 ng/mL were the most frequent category; however, markedly elevated CEA values were also observed in a subset of patients. Although this study did not evaluate survival outcomes, previous research has shown that elevated preoperative CEA levels are associated with advanced disease stage, poorer prognosis, and increased recurrence risk [8,9,13,14]. Most patients had CA 19-9 levels below 40 U/mL, whereas elevated values were observed in a smaller subset of patients. Given the descriptive nature of the present study and the incomplete availability of patient-level tumor marker data, no further comparative analysis between biomarker levels and clinical outcomes was performed.

This study has several limitations inherent to its retrospective observational design, including reliance on medical record documentation, the absence of long-term follow-up or survival data, and the relatively small sample size derived from a single-center experience. In addition, the incomplete availability of patient-level tumor marker data limited our ability to perform more detailed comparative analyses between biomarker levels and clinical outcomes. Comprehensive patient-level data on all metastatic sites were also not consistently available, limiting a broader characterization of metastatic patterns among patients with stage IV disease. These limitations should be taken into account when interpreting the findings.

Despite these limitations, our findings contribute valuable regional data regarding CRC characteristics and metastatic patterns. Strengthening screening programs and promoting early detection remain essential strategies to reduce the burden of advanced-stage disease and metastatic disease.

## Conclusions

Colorectal cancer in this study predominantly affected older male patients, with rectal tumors representing the most frequent primary location. A significant proportion of patients were diagnosed at stage III or IV, reflecting late-stage presentation. Hepatic metastasis occurred exclusively in patients with stage IV disease, consistent with the expected pattern of metastatic presentation.

These findings highlight the importance of early detection, systematic staging, and improved screening efforts to reduce advanced-stage diagnoses and metastatic disease burden. Further multicenter studies with larger sample sizes are warranted to validate these observations and strengthen regional epidemiological data.
